# Role of Resveratrol in Regulating Cutaneous Functions

**DOI:** 10.1155/2020/2416837

**Published:** 2020-04-14

**Authors:** Si Wen, Jiechen Zhang, Bin Yang, Peter M. Elias, Mao-Qiang Man

**Affiliations:** ^1^Dermatology Hospital, Southern Medical University, Guangzhou 510091, China; ^2^Department of Dermatology, Huadong Hospital, Fudan University, Shanghai 200040, China; ^3^Department of Dermatology, University of California San Francisco and Veterans Affairs Medical Center, San Francisco, CA 94121, USA

## Abstract

Protective role of the skin is against external insults and maintenance of electrolyte homeostasis of the body. Cutaneous dysfunction can account for the development of both cutaneous and systemic disorders. Thus, improvements in cutaneous functions can benefit a number of extracutaneous and cutaneous functions. Resveratrol, a natural ingredient, displays multiple benefits for various systems/organs, including the skin. The benefits of resveratrol for cutaneous functions include stimulation of keratinocyte differentiation and antimicrobial peptide expression, inhibition of keratinocyte proliferation and cutaneous inflammation, UV protection, anticancer, antiaging, and inhibition of melanogenesis. The mechanisms of action of resveratrol include activation of sirtuin 1 and nuclear factor erythroid 2-related factor 2, and inhibition of mitogen-activated protein kinase signaling. Evidence suggests that topical resveratrol could be a valuable alternative not only for daily skin care, but also for the prevention and treatment of various cutaneous disorders. This review summarizes the benefits of resveratrol for cutaneous functions.

## 1. Introduction

In the traditional view, the skin serves as a protective barrier between the body and the external environment. Yet, more and more evidence suggests that cutaneous function extends far beyond mere protection. In fact, cutaneous function regulates a wide spectrum of cutaneous and systemic functions. Compromised epidermal function has been linked to the development of a variety of cutaneous and extracutaneous disorders. For example, disruption of epidermal permeability barrier not only provokes the release and production of proinflammatory cytokines [1–3], but also induces infiltration and activation of inflammatory cells in the skin [4–7], suggesting that defective epidermal permeability barrier predisposes to the development of inflammatory dermatoses [8–11]. But barrier disruption also stimulates barrier homeostasis responses, including epidermal proliferation and lipid synthesis [12, 13]. Moreover, defects in epidermal permeability barrier allow the penetration of microbial pathogens into the skin [14–16].

Importantly, recent studies showed that the epidermal dysfunction-induced elevations in cutaneous cytokines lead to increased levels of proinflammatory cytokines not only in the skin, but also in circulation, supporting not only a pathogenic role for epidermal function in cutaneous and extracutaneous inflammation, but also suggesting a link between cutaneous function and inflammation-associated systemic disorders [17, 18]. In addition to epidermal permeability barrier homeostasis, other epidermal functions, such as pH and stratum corneum hydration, also regulate cutaneous functions [19–22]. Accordingly, improvements in epidermal function could benefit multiple cutaneous and extracutaneous functions [17, 23–25].

Because of the importance of cutaneous function, much recent attention has focused on the identification of active ingredients that could lead to the development of products that improve cutaneous function. In comparison with synthetic chemicals, natural ingredients are generally considered to be cheaper and more widely available, but still exhibit comparable benefits [26–28]. Among natural ingredients, bioflavonoids, including hesperidin, apigenin, epigallocatechin gallate, and resveratrol, exhibit a wide spectrum of biological activities, including antioxidative, anti-inflammatory, anticancer, antiaging, and UV protection, while improving cutaneous functions. In this article, we review the current in frontier about the regulatory role of resveratrol in cutaneous functions.

## 2. Properties and Sources of Resveratrol

**Figure 1:**
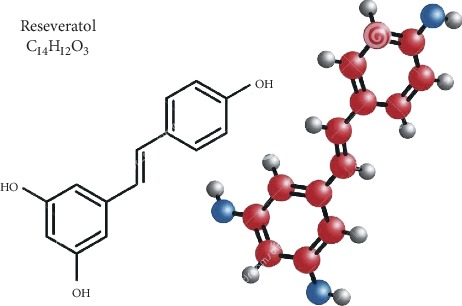
Structure of resveratrol.

**Figure 2:**
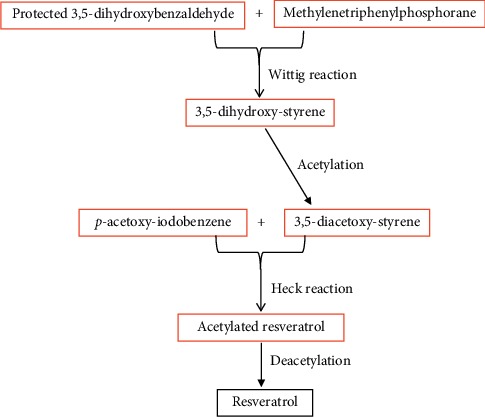
Synthesis of resveratrol [30].

**Figure 3:**
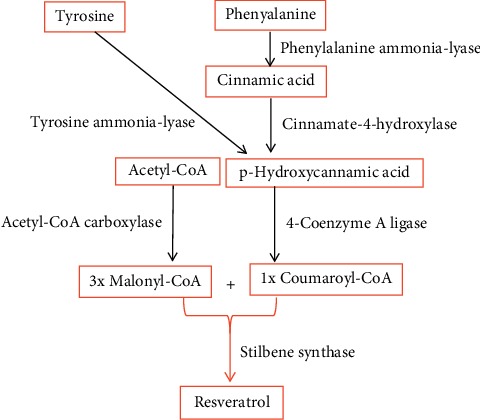
Biosynthesis of natural resveratrol.

Resveratrol (3,5,4′-trihydroxy-*trans*-stilbene) is a stilbenoid, belonging to a group of phytoalexins, with a molecular weight of 228.247 g/M and a melting point of 254°C (Figure 1 from http://www.streamstime.com), first identified in 1939 [29]. Resveratrol dissolves well in both ethanol and acetone (50 mg/ml), but barely dissolves in water (3 mg/1000 ml). It can be synthesized via Wittig reaction of protected 3,5-dihydroxybenzaldehyde and methylenetriphenylphosphorane, using reagent aldehyde : sodium hydride : CH_3_P(C_6_H_5_)Br at a ratio of 1 : 4 : 4 [30, 31] (Figure 2). Yields of resveratrol can be increased by using unprotected 3,5-dihydroxybenzaldehyde and aldehyde : sodium hydride : CH_3_P(C_6_H_5_)Br at a ratio of 1 : 4 : 4 [32]. In contrast, the biosynthesis of resveratrol in plants can begin with acetyl-CoA, tyrosine, or phenylalanine in response to external stressors, such as insults from insects, microbial pathogens, animals, or adverse weather conditions [33–35] (Figure 3). Resveratrol exists as both *trans* and *cis* isomers in plants. *Trans*-resveratrol can be isomerized to the *cis* form by UV irradiation [36].

Stilbene synthase is a key enzyme in the synthesis of resveratrol in the plants. Studies demonstrated that both expression levels of stilbene synthase and resveratrol content are regulated by a transcription factor, Myb14, which binds to Box L5 motif, leading to elevated stilbene synthase gene expression [37]. A number of other factors also regulate stilbene synthase expression and resveratrol production. For example, leaves contain the highest level, while the shoot tips have a relatively low level of stilbene synthase protein and mRNA in 1-year-old potted grapevines of *Vitis vinifera* L. cv. Cabernet Sauvignon [38]. Moreover, during grape development, expression levels of stilbene synthase mRNA increase continuously in the grape skin until they became ripe [39]. Likewise, old leaves of Cabernet Sauvignon exhibit higher levels of stilbene synthase mRNA than young leaves [39].

Infections of grapes can also change the expression of stilbene synthase mRNA. Dai et al. reported that incubation of cabernet sauvignon leaves with powdery mildew fungal spores for 24 hours significantly increased expression levels of stilbene synthase mRNA [39]. Additionally, irradiation of grape leaves with UVC not only increased the expression levels of both stilbene synthase protein and mRNA, but also increased resveratrol content, starting as early as 8 hours after irradiation [38]. In grape skin, UVB (312 nm) irradiation mainly increased expression levels of stilbene synthase mRNA in unripe grapes [40]. Wilting grapes at 28°C increased *trans*-resveratrol content, peaking at day 60, followed by a decline [40]. Incubation of grape leaves with 50 mM calcium chloride (CaCl_2_) for 24 hours induced greater than 2-fold increase in *trans*-resveratrol content in comparison with vehicle control [41]. Irradiation with UVC could further increase *trans*-resveratrol content in comparison with CaCl_2_ alone [41]. UVC irradiation primarily increased *trans*-resveratrol content in young grapes [42]. Seasonal changes in resveratrol content were also observed in grape roots, with a higher content (103–123 mg/kg dry weight) in the months of May and August to October, and a lower content (61–93 mg/kg dry weight) in the remaining months of the year [43]. Fungicides and methyl jasmonate treatments can markedly reduce resveratrol content in grapes and strawberry, respectively [44, 45]. Taken together, environmental conditions and the stages of plant development affect resveratrol synthesis and content.

Among plants, resveratrol is most abundant in the skin of red grapes [46]. But some other foods and beverages also contain fair amounts of resveratrol [34, 47–56]. Moreover, it appears that the age of the beverage determines its resveratrol content. For instance, 15-year-old champagne wine only contains 1 mg/L *trans*-resveratrol while the same wine, aged for 8 years, contains 45 mg/L *trans*-resveratrol [53]. Resveratrol content also varies with different parts of plants. The highest content of resveratrol was found in the stem phloems, but the lowest in the leaves in 1-year-old potted grapevines [38]. Likewise, resveratrol content in the grape skin is higher than that in the leaves and seeds for some grapes [57, 58]. But some grape seeds contain a higher content of resveratrol than do the skins [58]. Moreover, resveratrol content varies greatly with cultivars [33, 57–62]. For example, resveratrol content in small white Spanish peanuts is over 20-fold higher than that in White's runner peanuts [33]. Similarly, resveratrol content in the skin of rootstock grapes is over 100 times that in table grapes [57]. Moreover, resveratrol content in the skin of Berlandier Resseguier grapes is over 170 times of that in the skin of Dog Ridge grapes (366 vs. 20 *μ*g/g fresh weight) [57], the same as in tomatoes, in which the total resveratrol content is 18.4 *μ*g/g fresh weight in Micro Toms, and 0.34 and 0.38 *μ*g/g fresh weight, respectively, in Plum Toms and Ugly Ripes [63]. Thus, resveratrol content varies with the age of beverage, plant parts, and the cultivars.

Other factors, such as cultivation sites and nitrogen availability, can also influence the resveratrol content in plants [35, 62, 64, 65]. For example, the *trans*-resveratrol content in *V. vinifera* grape cane grown in Yantai is lower than that grown in Yangling, China (755 vs. 938 mg/kg fresh weight) [62]. Likewise, knotweed from Zunyi, China, contains 4.27 ± 0.09 mg/g of resveratrol. But knotweed from Yunnan only contains 2.06 ± 0.03 mg/g of resveratrol, while resveratrol was undetectable in knotweed grown in Dunstaffnage, Canada [65]. Similarly, the total resveratrol content is as high as 3.11 mg/L in merlot red wines made from grapes cultivated in Nagano, Japan, while the same wines only contain 0.88 mg/L of total resveratrol, if the grapes are cultivated in Iwate [35].

Variation of resveratrol content in plants is likely due to different environment/weather conditions [40–43, 64] and chemical compositions of soils [45, 66]. In addition, processing methods of foods and beverages can affect resveratrol content. Studies have shown that neither raw nor dry-roasted peanuts contain detectable *trans*-resveratrol [67, 68], but *trans*-resveratrol content was ≈1.6 mg/100g in boiled peanuts [67] and 10 to 50 *μ*g/100 g in peanut butter [68]. Moreover, preparation and/or processing methods can influence resveratrol content in beverages. For example, ultrasonic cleaning of grapes could increase resveratrol by as much as over 50% in comparison with that without ultrasonic cleaning [69]. Similarly, heating highbush Michigan blueberries at 190°C for 18 min reduced *trans*-resveratrol by over 40% as compared to raw blueberries [55]. Moreover, methanol extraction of grapes yields resveratrol as much as two times of acetone extraction [70]. In summary, plants and fruits contain a fair amount of resveratrol. But environmental conditions, developmental stage, and the parts of plants or fruits largely determine resveratrol content, while processing methods and storage conditions can affect the resveratrol content of foods and beverages. The content of resveratrol in certain foods, plants, and beverages is summarized in Supplemental [Supplementary-material supplementary-material-1].

## 3. Benefits of Resveratrol for Cutaneous Functions

The benefits of resveratrol for health have been demonstrated in multiple systems and/or organs, including cardiovascular system, diabetes, and immune as well as the neural system [71–76]. A large body of evidence, summarized here, has also indicated the benefits of resveratrol for the skin (Table 1) [77–126].

### 3.1. Keratinocyte Proliferation and Differentiation

Both keratinocyte proliferation and differentiation are required for the epidermis to reach its ultimate goal of the formation of the stratum corneum, an essential structure for epidermal permeability barrier. Several studies have demonstrated that resveratrol inhibits keratinocyte proliferation while stimulating differentiation. Resveratrol at a concentration as low as 2 *μ*M markedly inhibits DNA synthesis in keratinocyte cultures [77]. Inhibitory effect of resveratrol on DNA synthesis occurred as early as 24 hr after addition to cultures, with an IC50 range of 2–8 *μ*M [77]. Incubation of keratinocytes with resveratrol, at concentrations as low as 0.25 *μ*M for 72 hr, could induce a dose-dependent reduction in the number of living cells. Wu et al. reported that 20 *μ*M resveratrol inhibited keratinocyte proliferation by over 80%, using the 5-bromo-2-deoxyuridine assay [78]. With long-term treatments (e.g., two weeks), even 0.197 *μ*M resveratrol was sufficed to inhibit keratinocyte proliferation by 80% [82].

The activities of keratinocyte proliferation and differentiation are coordinated in an inverse manner, while terminal differentiation is crucial for the formation of permeability barrier. In contrast to proliferation, resveratrol stimulates keratinocyte differentiation. For example, treatment of keratinocytes with 3 *μ*M resveratrol until 3 days after postconfluence increased involucrin expression by 1.5-fold [83]. Moreover, formation of cornified envelopes, critical structures for epidermal permeability barrier, requires transglutaminase [127]. Treatment of keratinocytes with resveratrol induced a dose-dependent increase in transglutaminase activity, while simultaneously inhibiting DNA synthesis, with a half-maximal concentration of 35 *μ*M. Finally, resveratrol and 1, 25(OH)_2_D_3_ synergistically increased transglutaminase activity [84]. Thus, resveratrol inhibits keratinocyte proliferation, while stimulating differentiation.

### 3.2. Anti-UV Irradiation

While suberythemogenic doses of UVB irradiation instead enhance epidermal function, including improvements in epidermal permeability barrier function, stimulation of epidermal lipid synthesis and keratinocyte differentiation, and antimicrobial defense [128], excessive exposure to UV irradiation can damage the skin, causing sunburns, skin cancers, and photoaging. Both *in vivo* and *in vitro* studies have demonstrated that resveratrol protected the skin from UV irradiation. For example, addition of 0.1 *μ*M resveratrol to the culture medium immediately postirradiation provided 100% protection against UVA-induced reductions in keratinocyte proliferation and an increase in malondialdehyde (MDA) content. In parallel, both superoxide dismutase (SOD) and glutathione peroxidase (GSH-Px) contents in UVA-irradiated keratinocytes were normalized by treatment with resveratrol [85]. Either prior to or post-UVA irradiation, treatment of keratinocytes with resveratrol decreased lipid peroxidation. But only pretreatment, not posttreatment with resveratrol, increased SOD and glutathione S-transferase (GST) protein levels [86]. In addition to UVA irradiation, resveratrol can also protect keratinocytes from UVB-induced damages. For example, treatment of keratinocytes with resveratrol 1 hr prior to UVB irradiation decreased apoptosis rates by over 50% [87]. Moreover, treatment of keratinocytes with resveratrol prior to UV irradiation decreased the expression levels of proinflammatory cytokines, such as IL-6, IL-8, and TNF-*α*, by ≈50% from untreated keratinocytes [91]. Likewise, addition of 10 *μ*M resveratrol immediately after UVA and UVB irradiation also lowered the expression levels of IL-1-beta and IL-6 [93]. In addition, treatment of dermal fibroblasts with resveratrol after UVB irradiation decreased the contents of (i) reactive oxygen species, (ii) TNF-*α* and IL-6, (iii) inducible nitric oxide synthase (iNOS), and (iv) expression levels of matrix metallopeptidase I, while increasing procollagen 1 and elastin [94]. It appeared that pretreatment of keratinocytes with low concentration of resveratrol did not inhibit UV irradiation-induced inflammation [93].

The protective effects of resveratrol against UV irradiation have also been demonstrated *in vivo*. Afag et al. reported that topical application of 25 *μ*M resveratrol 30 min prior to UVB irradiation (180 mJ/cm^2^) decreased skin thickness and punch weight of ear by over 50 and 81%, respectively, in comparison with vehicle-treated controls [95]. Moreover, cutaneous inflammatory infiltration and ornithine decarboxylase protein levels decreased significantly in mouse skin treated with topical resveratrol prior to UVB irradiation [96]. Yet, topical applications of resveratrol post-UVB irradiation decreased whole skin thickness, but not epidermal thickness [97]. Some studies showed that resveratrol and UV irradiation synergistically enhanced expression levels of IL-8 mRNA and protein, and DNA damage in keratinocytes as compared to UV irradiation alone [129, 130]. These discrepant results could be attributable to variations, including the doses of resveratrol and UV irradiation, treatment timing, and/or the status of growth and differentiation in these cells. Thus, further studies are needed to clarify the effects of resveratrol on cutaneous function.

### 3.3. Antioxidant Defense

A number of factors, including psychological stress, cigarette smoke, air pollution, and UV irradiation, can cause oxidative stress, contributing to the development of aging and a line of disorders such as dermatoses, inflammation, cardiovascular diseases, cancer, and neurodegenerative diseases [131–139]. Accordingly, antioxidants have been utilized to prevent and treat certain disorders [140–142]. Among the antioxidants, benefits of resveratrol have also been studied extensively both *in vitro* and *in vivo*. Phase II enzymes, including GSH-Px, quinone dehydrogenase (NQO), GST, SOD, and GSH, are responsible for antioxidation in living organisms. Soeur et al. reported that 10 *μ*M resveratrol induced an over 2-fold increase in expression levels of mRNA for GSH-Px and NQO1, along with over 1-fold increase in glutathione synthesis, in keratinocytes, in addition to an over 7-fold increase in expression levels of mRNA for GSH-Px and NQO1 in reconstructed human skin [99]. Resveratrol can also protect keratinocytes from damages caused by oxidative stressors, including nitric oxide (NO), H_2_O_2_, cigarette smoke, and arsenic [92, 101–105]. Surprisingly, topical resveratrol did not mitigate UVB irradiation-induced reductions in both activity and expression levels of antioxidant enzymes, including catalase, SOD, and GSH-Px, in a mouse model of chronic UVB irradiation [97]. NO is a free radical required for maintenance of normal function, but excessive NO can induce oxidative stress, leading to apoptosis [136]. Topical applications of human skin with NO donor (nitrite) caused cutaneous inflammation and keratinocyte apoptosis [137]. Likewise, 3 mM sodium nitroprusside induced ≈70% inhibition of keratinocyte proliferation [100]. Treatment of keratinocytes with the combination of 30 *μ*M resveratrol and 3 mM sodium nitroprusside reduced caspase 3 and 9 activity as well as apoptotic rate by 80% from cultures treated with sodium nitroprusside alone. H_2_O_2_ is commonly used as stressor to induce oxidative stress. Following the treatment of keratinocytes with 400 *μ*M H_2_O_2_, reactive oxygen species (ROS) content increased 1.5-fold over the controls [92]. Resveratrol at concentrations of 25 *μ*M and 100 *μ*M lowered ROS contents by 25% and 40%, respectively, in comparison with cells treated with H_2_O_2_ alone. Finally, the protective effects of resveratrol against oxidative stress have also been demonstrated in keratinocytes treated with either arsenic or cigarette smoke [103–105].

The skin, an interface between the body and external environment, is vulnerable to environmental insults, including oxidative stress, leading to acceleration of skin aging and the development of a variety of skin disorders [138, 139, 143]. Therefore, enhancement of antioxidant defense of the skin could help manage various cutaneous dermatoses. Krajka-Kuźniak et al. showed that a single topical treatment of mouse skin with 16 *μ*M resveratrol for 4 hr increased GST activity by 63% and GST content by 22% over controls [98]. Similarly, a greater than 1-fold increase in GST activity was observed in mouse epidermis 24 hr after topical application of 16 *μ*M resveratrol [106]. Likewise, production of free radicals was reduced in human stratum corneum following topical treatment with resveratrol for 24 hr [107]. Collectively, resveratrol exhibits antioxidant properties both *in vitro* and *in vivo*.

### 3.4. Anticancer

Oxidative stress has been considered as a major contributor to the development of skin cancers, such as melanoma [144–146]. Accordingly, antioxidants, including resveratrol, have been used to prevent and treat skin cancer [147–149], although exacerbation and increased risk of cancers have been reported [150, 151]. Melanoma is the most serious type of skin cancer, with mortality rates of 3.1/100,000 in the US [152] and 1.4–1.9/100,000 in Germany [153]. Antimelanoma benefits of resveratrol have been demonstrated both *in vivo* and *in vitro*. Studies in melanoma cell cultures showed that resveratrol inhibited proliferation of melanoma cells, with a concentration of 7 *μ*g/ml inducing a 50% inhibition of cell growth [108]. It seems that melanoma cells are more sensitive to resveratrol than keratinocytes, which instead required a concentration of 20 *μ*g/ml to cause 50% inhibition of cell growth. Moreover, resveratrol dose-dependently increased both apoptotic and necrotic cells after 24 hr treatment of two melanoma cell lines [108]. In addition, resveratrol also inhibited the growth of squamous cell carcinoma cells (A431 cell line) [81]. Resveratrol-induced apoptosis and inhibition of cell proliferation were also observed in human head and neck squamous cell carcinoma cell lines, e.g., FaDu, Cal27, and Det562 cell lines [111]. Moreover, rates of DNA synthesis were reduced by 78% following the treatment of carcinoma cells with 25 *μ*M resveratrol for 72 hr [109]. *In vivo* studies showed that oral administration of resveratrol resulted in a dose- and time-dependent inhibition of carcinoma cell growth, with over 50% reductions in both tumor volume and weight per mouse following 30-day treatment with resveratrol at a dose of 50 mg/kg body weight [111]. Taken together, resveratrol demonstrates potential anticancer activity both *in vivo* and *in vitro*.

### 3.5. Anti-Inflammation

Inflammatory dermatoses prove the most common clinical problems in dermatology. Because of the severe side effects of immune modulators, such as glucocorticoids and tacrolimus, safe and effective alternatives are highly demanded. Studies have shown that resveratrol is safe in both animals and humans [154, 155], but it can effectively inhibit inflammation both *in vitro* and *in vivo*. For example, incubation of keratinocytes with 50 *μ*M resveratrol for 3 hr lowered the expression levels of mRNA for IL-6, IL-8, TNF-*α*, and macrophage inflammatory protein 1 (MIF-1) by over 50% [91]. While production of proinflammatory cytokines markedly increased in keratinocytes exposed to TNF-*α*, lipopolysaccharide, interferon *γ* (IFN-*γ*), and UV irradiation [91, 102, 112–114], addition of 50 *μ*M resveratrol 1 hr prior to lipopolysaccharide treatment dramatically decreased mRNA levels (>40% reduction) for MIF-1, IL-6, and cyclooxygenase 2 in comparison with keratinocytes treated with lipopolysaccharide alone [91]. Yet, resveratrol also dose-dependently stimulated IL-8 production in keratinocyte cultures [91], suggesting that resveratrol differentially regulates cytokine production.

Although there is still little or no evidence that resveratrol can inhibit cutaneous inflammation in humans, anti-inflammatory benefits of resveratrol have been well demonstrated in murine models of inflammatory dermatoses. For example, in acute allergic contact dermatitis model, pretreatment of mouse ears with resveratrol reduced the density of CD3^+^ cells by≈90% in parallel with significant reduction of ear thickness and expression levels of intercellular adhesion molecule 1 (ICAM-1), C-X-C motif chemokine ligand 10 (CXCL10), C-C motif chemokine ligand 2 (CCL2), and IFN-*κ* in the epidermis [113], suggesting preventive benefits of topical resveratrol in acute allergic contact dermatitis. Moreover, the therapeutic effects of topical resveratrol have also been demonstrated in an atopic dermatitis-like disease model. Kang et al. showed that topical applications of either 2.5% resveratrol or resveratrol-enriched rice extract for five weeks markedly reduced dermatitis score and serum IgE levels in a dermatitis model induced by topical dinitrochlorobenzene [114]. Likewise, both intravenously and orally given resveratrol significantly alleviated dermatitis score and decreased cytokine expression [117, 118].

Psoriasis is another common, inflammatory skin disorder. Kjaer et al. showed that oral administrations of *trans*-resveratrol at a daily dose of 400 mg/kg body weight induced over 50% reductions in both erythema and scale scores, along with 15% decrease in skinfold thickness of the back skin, in an imiquimod-induced psoriasis-like mouse model [119]. In addition, topical resveratrol also decreased the expression levels of IL-17a and IL-19 mRNA by ≈60% in comparison with mice treated with imiquimod alone [119]. Together, these results show that both topical and systemic administration of resveratrol can mitigate cutaneous inflammation.

### 3.6. Cutaneous Wounding

Cutaneous wound healing involves the proliferation of both fibroblast and keratinocytes, as well as collagen deposition. A number of observations suggested that, in murine models of full-thickness wound, resveratrol stimulates cell proliferation and collagen, leading to acceleration in cutaneous wound healing. Application of wound dressings containing resveratrol to full-thickness skin wounds induced marked reductions in wound areas in comparison with the controls [122]. Similar acceleration in wound closure occurs following the placement of scaffolds containing resveratrol over the wound [123]. This evidence indicates that topical resveratrol accelerates wound healing in normal mice.

Management of slow wound healing in diabetics has been a substantial challenge. Yes, studies have shown that either topical or systemic administrations of resveratrol can improve cutaneous wound healing in animal model of diabetes. Moreover, the efficacy of topical resveratrol ointment for wound healing was superior to that of topical *β*-sitosterol, conventional wound healing product [124]. Huang et al. also reported that a single application of resveratrol accelerated cutaneous wound healing in diabetic mice [125]. However, other studies have shown little or no benefit of resveratrol in wound healing following a single application in diabetic rats [126]. Thus, the potential benefits of resveratrol for wound healing in diabetic models still need to be validated.

### 3.7. Others

Studies in both humans and murine models reveal that resveratrol also regulates other cutaneous functions, including skin aging, melanogenesis, and antimicrobial defense. In human keratinocyte cultures, resveratrol reduced 90% reduction in expression levels of beta-galactosidase, a biomarker of senescence, in an aging model induced by oxidative stress [156, 157]. Clinical trials have also demonstrated the antiaging properties of resveratrol. A study in 50 humans with clinical signs of aging showed that oral fruit extracts that contain resveratrol markedly improved multiple aging-associated parameters, including increased stratum corneum hydration and skin elasticity, decreased skin roughness and wrinkle depth, as well as reductions in the intensity of pigmented solar lentigines [158]. In parallel, levels of plasma derivatives of ROS dramatically declined, while skin ferric-reducing ability increased. In addition, topical applications of resveratrol-containing products also improved aging-associated signs, such as skin wrinkles, stratum corneum hydration, and pigmentation, in aged humans [159]. But in one clinical trial in 30 subjects, oral supplement of product containing *trans*-resveratrol did not appreciably improve skin aging, despite reductions in cutaneous MDA content and elevations in SOD content [160]. These discrepant results suggest that additional trials are still needed to determine whether resveratrol benefits skin aging.

Other studies suggest that resveratrol exhibits antimicrobial properties. Cathelicidin antimicrobial peptides (CAMP) are a family of polypeptides, produced by keratinocytes, macrophages, and polymorphonuclear leukocytes, that display antibacterial, antifungal, and antiviral activities. Park et al. reported that incubation of keratinocytes with resveratrol for 24 hours increased the expression level of CAMP mRNA by over 4-fold [161]. But resveratrol may also directly inhibit microbial growth because incubation with resveratrol induced time- and dose-dependent reductions in Propionibacterium acnes colony-forming units, possibly due to disruption of the bacterial membrane [162]. Studies have shown that resveratrol exhibits several bactericidal and bacteriostatic activity against several pathogens, including *S. pyogenes*, *S. aureus*, *C. glabrata*, *and C. albicans*, with minimum inhibitory concentrations as low as 1.25 mg/ml [163]. Moreover, topical applications of 25% resveratrol cream markedly decreased lesion scores for herpes simplex infection, with an efficacy comparable to 5% acyclovir ointment, in a mouse model of herpes simplex infections [164]. Similar results were also obtained with topical applications of oxyresveratrol in mice infected by herpes simplex virus [165, 166]. Furthermore, studies also suggest benefits of resveratrol for keloids. For example, resveratrol induced apoptosis of fibroblasts from keloids, in parallel with reductions in the expression levels of mRNA for collagen 1 and procollagen 3, while increasing expression of SIRT1, suggesting a potential application of resveratrol for the treatment of keloids and hypertrophic scars [167–169]. Other studies have demonstrated that topical resveratrol improves epidermal permeability barrier function and stratum corneum hydration in sodium dodecyl sulfate-damaged human skin [170]. Additionally, both *in vitro* and *in vivo* studies have shown that resveratrol reduces skin pigmentation via inhibition of tyrosinase activity, cytokine production, and melanocytic microphthalmia-associated transcription factor (MITF) expression [171]. Yet, all of these putative benefits of resveratrol for cutaneous function still lack sufficient clinical validation. Therefore, well-designed clinical trials are still required before resveratrol can be widely utilized in clinical settings.

## 4. Mechanisms of Action

**Figure 4:**
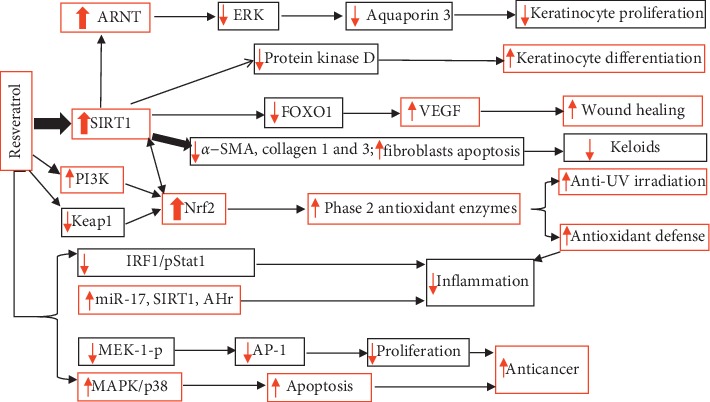
Schematic diagram of the mechanisms by which resveratrol regulates cutaneous functions. *α-*SMA: *α-*smooth muscle actin.

Evidence of resveratrol for multiple cutaneous functions has been well demonstrated, but the underlying mechanisms whereby resveratrol acts remain unclear. A line of evidence suggests that the actions of resveratrol could be via multiple mechanisms such as upregulation of nuclear factor erythroid 2-related factor 2 (Nrf2), activation of sirtuin 1 (SIRT1), and mitogen-activated protein kinase (MAPK) signaling pathway, depending on which function is regulated. The major putative mechanisms by which resveratrol regulates cutaneous function are illustrated in Figure 4.

### 4.1. Keratinocyte Proliferation and Differentiation

Keratinocyte proliferation and differentiation, which are inversely regulated, are both required to form the *stratum corneum*, the outmost layers of the skin, providing multiple cutaneous protective functions because resveratrol can stimulate keratinocyte differentiation while inhibiting proliferation, resulting in acceleration of epidermal maturation. The inhibitory effects of resveratrol on keratinocyte proliferation occur via two mechanisms: (i) activation/upregulation of SIRT1 and (ii) inhibition of protein kinase D. In keratinocyte cultures, resveratrol upregulated expression of SIRT1, leading to elevation in aryl hydrocarbon receptor nuclear translocator (ARNT), resulting in downregulation of aquaporin 3, and consequently inhibiting cell proliferation [78]. Lee et al. showed that resveratrol increased the expression level and deacetylase activity of SIRT1, resulting in apoptosis [172]. Other studies suggest that resveratrol inhibits DNA synthesis, while increasing transglutaminase activity via inhibition of protein kinase D activity [84]. Moreover, activation of SIRT1 by resveratrol could also increase keratinocyte differentiation [83]. Activation of SIRT1 by resveratrol is likely via enhancement of the binding of specific substrates to SIRT1 [173]. Thus, resveratrol could inhibit keratinocyte proliferation and stimulate differentiation via both activation of SIRT1 and/or inhibition of protein kinase D.

### 4.2. Protection from UV Irradiation and Antioxidant Defense

Although the precise mechanisms by which resveratrol protects the skin against UV irradiation and oxidative stress are unclear, a handful of evidence points to a central role of Nrf2. This transcription factor regulates phase 2 antioxidant enzymes, which protect against UV irradiation- and other oxidative stress-induced damage to the skin. Nrf2 deficiency accelerated UV irradiation-induced photoaging and inflammation [174, 175], while conversely activation of Nrf2 protects against UV irradiation-induced apoptosis and inflammation [176, 177]. Normally, Nrf2 together with Kelch ECH associating protein 1 (Keap1) forms a complex, which is degraded by the ubiquitin-proteasome system [178]. Upon oxidative stress (including UV irradiation-induced oxidative stress), Nrf2 is released from Nrf2/Keap1 complex and translocates into the nucleus, where Nrf2 binds to antioxidant response element (ARE) in a heterodimeric complex, consequently leading to increased production of phase 2 antioxidant enzymes. While UV irradiation can increase the production of reactive oxygen species and oxidative products [179, 180], resveratrol can attenuate UV-induced oxidative stress via upregulation and/or activation of Nrf2. For example, treatment of keratinocytes with resveratrol either before or after UVA irradiation induced >50% increase in Nrf2 content, while increasing content of Nrf2 in the nuclear fraction [86, 90]. Similarly, treatment of either normal mice or oxidative-stressed keratinocytes with resveratrol increases Nrf2 expression and activation [98, 99], leading to increased expression of phase 2 antioxidant enzymes and reductions in reactive oxygen species [85, 90, 100], ultimately protecting/alleviating cell damage induced by UV irradiation or other oxidative stressors.

With regard to how resveratrol upregulates Nrf2 expression and activity, at least three mechanisms probably are operative. One mechanism involves upregulation of SIRT1 expression. Resveratrol is a SIRT1 activator [83]. Treatment of adipocytes with resveratrol significantly increased expression levels of SIRT1 mRNA [181]. Upregulation of SIRT1 expressions, in turn, increases expression levels of Nrf2 and phase 2 antioxidant enzymes, while silencing SIRT1 with siRNA decreases Nrf2 protein as well as activity of ARE promotor [182]. Moreover, upregulation and activation of Nrf2 expression by SIRT1 were also observed [183]. The second mechanism comprises direct upregulation of Nrf2 expression because studies have shown that resveratrol increases Nrf2 expression in kidney, heart, and lung tissues [184, 185]. The third mechanism is direct downregulation of Keap1 expression. Treatment of keratinocytes with resveratrol either before or post-UVA irradiation lowers expression levels of Keap1 protein [86]. Resveratrol-induced reduction of Keap1 expression was also observed in the kidney and lung tissues of obese and asthmatic rats [185]. Reductions in Keap1 expression can slow Nrf2 degradation, resulting in an increase in Nrf2 expression. Other studies also showed that resveratrol stimulated Nrf2 expression and nuclear translocation without changing the expression levels of Keap1 [98, 184]. Interestingly, Nrf2 and SIRT1 can positively coregulate each other. For example, treatments of either renal tubular cells or glomerular mesangial cells with resveratrol parallelly increased Nrf2 and SIRT1 expression. Knockdown of Nrf2 with siRNA decreases the expression levels of SIRT1, and vice versa [184, 186]. Therefore, resveratrol can sequentially or separately upregulate SIRT1 and Nrf2, while downregulation of Keap1 expression, resulting in increased expression of phase 2 antioxidant enzymes, which in turn protect the skin from UV irradiation- and oxidative stress-induced damage. Finally, one study showed that inhibition of phosphatidylinositol-3-kinase prevented the activation of Nrf2 induced by pterostilbene, a resveratrol analog, suggesting that resveratrol can also activate Nrf2 via activation of phosphatidylinositol-3-kinase [90].

Still other mechanisms could also account for the actions of anti-UV irradiation and antioxidative stress. For example, pretreatment of keratinocytes with resveratrol almost completely prevents the activation of NF*κ*B induced by UVB irradiation [89], suggesting that resveratrol-induced inhibition of NF*κ*B activation could contribute to its anti-UV irradiation properties. Resveratrol-induced upregulation of heat-shock protein 27 (HSP27) and downregulation of caspase 3 could also contribute to its anti-UV irradiation property [187]. Thus, resveratrol protects skin against UV irradiation and oxidative stress via multiple mechanisms.

### 4.3. Anticancer Activity

Both *in vitro* and *in vivo* studies have shown that resveratrol also inhibits proliferation, while stimulating apoptosis of cancer cells via several mechanisms. First, resveratrol induces apoptosis and phosphorylation of MAPK/ERK and MAPK/p38 in addition to increasing expression levels of caspase 3 and p53, while conversely, inhibition of p38 abolished its apoptotic effects [81, 188]. It appears that resveratrol-induced phosphorylation of p53 and apoptosis is mediated by c-Jun NH2-terminal kinases because knockdown of c-Jun NH2-terminal kinase genes prevented both phosphorylation of p53 and apoptosis induced by resveratrol [189]. Therefore, resveratrol-induced activation of the MAPK/p38 signaling pathway likely accounts, at least in part, for its anticancer effects. Regarding antiproliferation of cancer cells, resveratrol inhibits expression of MEK1-P and ERK1/2-P, leading to reductions in cyclin D1 and cyclin-dependent kinase 6 expression, resulting in cell cycle at rest [109]. Moreover, resveratrol also decreased c-Jun levels and reduced DNA-binding and transcriptional activity of activator protein-1, which is required for initiation of DNA synthesis [190]. Other studies showed that inhibition of NF-*κ*B, cyclooxygenase 2, phosphatidylinositol-3-kinase, and P450 isoenzyme CYP1A1 and induction of caspases 3 and 9 also could contribute to anticancer effects of resveratrol [191–196]. Thus, resveratrol-induced reductions in expression levels of MEK1-P and ERK1/2-P and decreased activator protein-1 activity could contribute to its inhibition of cancer cell proliferation.

### 4.4. Anti-Inflammatory Activity

The anti-inflammatory effects of resveratrol have been demonstrated in various *in vivo* and *in vitro* models, but the mechanisms of the actions of resveratrol are often unclear, depending on the inflammatory models employed in the studies. One possible mechanism is inhibition of NF-*κ*B signaling pathways. Activation of NF-*κ*B can upregulate transcription of cytokines while I*κ*B inhibits NF-*κ*B activity [102, 197]. Hence, degradation of phosphorylated I*κ*B would increase NF-*κ*B activity. Resveratrol-containing mixture inhibited I*κ*B phosphorylation and decreased NF-*κ*B, resulting in reductions in cytokine production in keratinocytes stimulated by TNF-*α* [102]. Another study suggests that inhibition of cytokine production by resveratrol seems linked to upregulation of miR-17 expression in keratinocytes stimulated with lipopolysaccharide because inhibition of miR-17 overcame the inhibitory effects of resveratrol on inflammation [112]. But one study showed that resveratrol increases IL-8 production in keratinocytes stimulated with a combination of TNF-*α* and IFN*γ* via upregulation of aryl hydrocarbon receptor expression [91]. Inhibition of allergic contact dermatitis by resveratrol could be attributable to the downregulation of interferon regulatory factor 1/STAT1 signaling pathway and inhibition of phosphorylation of MAPK/p38 and/or phospholipase C*γ* [113, 116, 198]. Moreover, resveratrol inhibited proliferation and differentiation of CD+ T cells and proliferation of Th17 T cells via upregulation of phosphorylated MAPK and downregulation of phosphorylated mammalian target of rapamycin (p-mTOR) in Jurkat cells [199]. Furthermore, resveratrol-induced inhibition of TNF-*α*-induced cytokine production in fibroblasts is via activation of SIRT1 because knockdown of SIRT1 abolishes the inhibitory effect resveratrol on inflammation [200]. Thus, resveratrol can inhibit cutaneous inflammation via a variety of mechanisms, including inhibition of NF-*κ*B, MAPK/p38, phospholipase C*γ*, and p-mTOR, upregulation of miR-17, and activation of SIRT1.

### 4.5. Wound Healing

Cutaneous wound healing is a complex process that can be accelerated by resveratrol via stimulation of neovascularization, keratinocyte differentiation, permeability barrier maturation, and antimicrobial activity. One study showed that resveratrol accelerates cutaneous wound healing and vascularization in aged rats through upregulation of SIRT1 and adenosine monophosphate-activated protein kinase (AMPK) pathway [121]. The role of SIRT1 signaling in vascularization has also been demonstrated in cutaneous wounds of diabetic mice. Topical applications of resveratrol to the wounded area of diabetic mice stimulated proliferation and inhibited apoptosis of endothelial cells, leading to accelerated wound healing, while either SIRT1 inhibitor or knockout of SIRT1 abolished the benefits of resveratrol in wound healing [125]. SIRT1-mediated benefits of resveratrol in diabetic wound healing can also be attributable to protection of endothelial cells from oxidative stress [201]. In addition, studies in mice indicate that resveratrol accelerates cutaneous wound healing by upregulation of vascular endothelial growth factor mediated by activation of at least two antioxidant enzymes (thioredoxin-1 and heme oxygenase-1) [123]. Because wound infections are the major cause of delayed wound healing [202], the antimicrobial properties of resveratrol could be another mechanism whereby wound healing is accelerated. Lastly, the ultimate goal of cutaneous wound healing is the formation of intact permeability barrier, which requires both lipid production and keratinocyte differentiation. Thus, resveratrol could also accelerate cutaneous wound healing through its well-known ability to stimulate keratinocyte differentiation and lipid production [83, 84, 161]. Collectively, the resveratrol-induced acceleration of cutaneous wound healing can be attributable to activation of SIRT1 and AMPK signaling, antioxidative stress, and enhanced formation of epidermal permeability barrier.

### 4.6. Others

The mechanisms whereby resveratrol induces apoptosis and inhibition of fibroblasts include inhibition of hypoxia-inducible factor 1, in which activation stimulates fibroblast proliferation while inhibiting apoptosis [203], downregulation of transforming growth factor *β*1 [169], miR-17 [204], as well as expression levels of mRNA for collagen 1 and procollagen 3 [167], whereas resveratrol-induced upregulation of antimicrobial peptides is via enhancing expression of sphingosine-1-phosphate, leading to activation of N-F*κ*B-C/EBP*α* signaling pathway [161].

Resveratrol inhibits melanogenesis by at least four different mechanisms: (1) in human melanocyte cultures, resveratrol inhibited tyrosinase synthesis and activity along with accelerated transport of newly synthesized tyrosinase to proteasomal complex, without dramatic alterations in mRNA levels of either melanocytic microphthalmia-associated transcription factor (MIFT) or tyrosinase [205]; (2) in melan-A cells, inhibition of melanogenesis by resveratrol is via induction of autophagy, leading to reduction in *α* melanocyte-stimulating hormone levels [206]. The latter stimulates melanin production and release via activation of melanocortin-1 receptor. Deletion of autophagy-related genes could prevent resveratrol-induced reduction in melanogenesis; (3) in human melanocyte cultures, resveratrol activated c-Jun N-terminal kinase, resulting downregulation of MITF expression [207]; and (4) anti-inflammatory effects of resveratrol can be an additional mechanism contributing to decreasing pigmentation [171]. The antioxidant properties of resveratrol largely account for its antiaging effects [208].

## 5. Additional Points

Both *in vitro* and *in vivo* studies showed that resveratrol regulates multiple cutaneous functions, including UV protection, anti-inflammation, antioxidant defense, acceleration of wound healing, antimicrobe, anticancer, and inhibition of melanogenesis. Upregulation of SIRT1 largely accounts for the mechanisms of resveratrol action although various other mechanisms are also involved. Thus, resveratrol benefits multiple cutaneous functions through a variety of mechanisms. While clinical data have revealed the benefits of resveratrol for extracutaneous systems/organs, including improvements in bone density, osteoarthritis, renal function, and diabetes, evidence is still insufficient to conclude its benefits for cutaneous functions in clinical settings. Therefore, proper clinical trials are still required to validate the clinical significance of the utility of resveratrol in the skin.

## Figures and Tables

**Table 1:** Benefits of resveratrol for cutaneous functions.

Models	Treatments	Benefits	Mechanisms	Ref.
*Keratinocyte proliferation/differentiation*				
Keratinocytes	Cells cultured with 0.25–100 *μ*M resveratrol for 24–72 hr	↓Proliferation	ND	[77]
	Cells cultured with 20 or 40 *μ*M resveratrol for 24 hr	↓Proliferation	↑SIRT1	[78]
	Cells cultured with 50 *μ*M resveratrol for 12 hr	↓Proliferation	ND	[79]
	Cells cultured with 25–100 *μ*M resveratrol for 24 hr	↓Proliferation	ND	[80, 81]
	Cells treated with 0.197 *μ*M resveratrol for 2 weeks	↓Proliferation	ND	[82]
	Cells cultured with 3 *μ*M resveratrol until 3 days postconfluence	↓Differentiation	↑SIRT1	[83]
	Cells cultured with 100 *μ*M resveratrol for 24 hr	↓Proliferation ↓Differentiation	↓Protein kinase D	[84]

*Anti-UV irradiation*				
Keratinocytes	Immediately after irradiation with UVA (5 J/cm^2^), cells were treated with 0.01–0.1 mM resveratrol for 24 hr	↑Cell viability ↓MDA content	↑SOD and GSH-Px expression	[85]
	Either before or after irradiation with UVA (2.796 J/cm^2^), cells were treated with 2.5 and 5.0 mg/l resveratrol, respectively	↑Cell viability ↑SOD and GST	↑NRF2 in nuclear translocation ↓Keap1	[86]
	Cells first treated with 10 *μ*M resveratrol for 1 hr, followed by UVB irradiation (30 mJ/cm^2^)	↑Cell viability ↓Apoptosis	↑Activation of SIRT1	[87]
	Cells first treated with 2% of resveratrol for 2 hr, followed by UVB irradiation (5–100 mJ/cm^2^)	↑Cell viability	ND	[88]
	Cells treated with 5–25 *μ*M resveratrol for 24 hr, followed by irradiation with UVB (40 mJ/cm^2^)	↓NF-*κ*B content and activity	↑I*κ*B*α* ↓IKK*α*	[89]
	Cells treated with 5–10 *μ*M pterostilbene, analog of resveratrol, for 24 hr, followed by irradiation with UVB (30 mJ/cm^2^).	↑Cell viability ↓ROS ↓DNA damage	↑Nrf2	[90]
	Cells treated with 50 *μ*M resveratrol 30 min prior to irradiation with 1 J/cm^2^ UVA +0.1 J/cm^2^ UVB	↓IL-6, MCP-1, and TNF-*α* mRNA	↑ARH	[91]
	Cells treated with 25 or 100 *μ*M resveratrol for 2 or 24 hr, followed by irradiation with UVB (10, 20, 40, or 100 mJ/cm^2^)	↑ROS ↓Autophagy	↑ERK activation ↑Bax/Bcl2 ratio	[92]
	Cells first irradiated with 1 J/cm^2^ UVA +0.1 J/cm^2^ UVB, followed by treatment with 10 *μ*M resveratrol.	↓CYP1A1, CYP1B1, IL-1*β*, IL-6, and COX2 mRNA levels	↓Peroxide content	[93]
Dermal fibroblasts	Immediately after UVB irradiation (144 mJ/cm^2^), fibroblasts were treated with 10 or 100 *μ*g/L of resveratrol-enriched rice extract at various concentrations for 24–72 hr	↑Cell viability ↓ROS ↓MMP1, p53, Bax, TNF-*α*, IL-6, iNOS, and COX2 ↑PIP1 and TGF-*β* protein	ND	[94]
Reconstructed human skin	Reconstructed human skin was treated with 1% of resveratrol-enriched rice extract for 24 hr, followed by irradiation with UVB (100 mJ/cm^2^)	↓MMP1 ↑PIP1, type I procollagen, and collagen fibers	ND	[94]
Mice	Mice were treated topically with 25 *μ*M resveratrol in 200 *μ*l acetone 30 min prior to irradiation with 180 mJ/cm^2^ UVB	↓Ear weight and edema ↓Inflammatory infiltrate ↓ODC activity and COX2 activity ↓Lipid peroxidation	ND	[95]
	Mice were treated topically with 10 *μ*M resveratrol in 200 *μ*l acetone 30 min prior to irradiation with 180 mJ/cm^2^ UVB	↑Cell proliferation ↓COX2 and ODC expression	↓Survivin	[96]
	Mice were treated topically with 0.48% resveratrol 20 min prior to irradiation with 360 mJ/cm^2^ UVB	↓Skin edema in mice treated with resveratrol either before or after UVB irradiation ↓Epidermal thickness in mice treated with resveratrol before UVB irradiation	↑NRF2	[97]
	Mice were treated topically with 0.48% resveratrol 20 min prior to irradiation with 180 mJ/cm^2^ UVB, 3 irradiation/week for a total of 30 weeks	↓Lipid, DNA, and protein peroxidation ↑Activity and expression of antioxidant enzymes		[97]

*Antioxidant defense*				
Keratinocytes	Cells cultured with 20 and 60 *μ*M resveratrol for 24 hr	↑GST activity	↑NRF2 expression and activation	[98]
	Cells pretreated with 10 or 20 *μ*M resveratrol for 16 hr	↑NQO1 and GSH-Px mRNA ↑GSH protein synthesis	↑NRF2 activation	[99]
	Cells treated with both 0.3–3 mM sodium nitroprusside and 1–30 *μ*M resveratrol for 24 hr	↑Cell viability ↓Caspase 3 and 9 activity	↓IL-8, NOS3, and NADPH dehydrogenase mRNA ↑GSH-Px mRNA	[100]
	Cells pretreated with 25 or 100 *μ*M resveratrol 24 hr prior to addition of 200, 400, or 800 *μ*M H_2_O_2_ and cells were harvested 48 hr postaddition of H_2_O_2_	↓ROS	ND	[92]
	Cells pretreated with 140 *μ*M resveratrol for 1 h, then 500 *μ*M H_2_O_2_ was added, and incubated for additional 2–16 h	↓DNA damage and HSP70 expression	ND	[101]
	Cells treated with 10 *μ*M resveratrol for 24 hr, and then 100 *μ*M H_2_O_2_ was added and incubated for additional 30 min	↓ROS ↓MDA	ND	[102]
	Cells pretreated with 0.5–10 *μ*M resveratrol for 24 hr, followed by removal of media, and then exposed to cigarette smoking for 50 min	↑Scavenger receptor class B type I protein and mRNA ↓4-Hydroxynonenal adducts	ND	[103]
	Cells pretreated with 10 *μ*M resveratrol for 24 hr, followed by removal of media, and then exposed to cigarette smoking for 50 min	↓ROS and carbonyl groups	↑Methionine sulfoxide reductase A mRNA ↓Transient receptor potential cation channel subfamily A member 1 mRNA and protein	[104]
	Cells pretreated with 0.5 *μ*M resveratrol for 3 hr, followed by addition of 0.1–20 *μ*M arsenic and incubation for additional 20 hr	↓Arsenic-induced increase in metabolic activity and expression of DNA polymerase beta ↑Arsenic-induced reduction in Y419 phosphorylation and Src protein	ND	[105]
Reconstructed human skin	Keratinocytes were pretreated with 20 or 100 *μ*M resveratrol for 16 hr, followed by removal of media, and then exposed to 800 *μ*M cumen hydroperoxide for 8 hr	↑GSH expression ↓Apoptosis	↑NRF2 activation	[99]
Mice	Mice were treated topically with 16 *μ*M resveratrol, and four hours later, skin samples were collected	↑GST activity and content	↑NRF2 activation	[98]
	Mice were treated topically with 8 or 16 *μ*M resveratrol, and twenty-four hours later, skin samples were collected	↑Glucuronosyltransferase and NADPH:quinone oxidoreductase activity	ND	[106]
Humans	Stratum corneum was collected with tape strip 24 hr after single application of resveratrol at a dose of 537 *μ*g/cm^2^ on the ventral forearm	↓Production of free radical	ND	[107]

*Anticancer*				
Melanoma cell line	Cells were treated with 20–40 *μ*g/ml resveratrol for up to 5 days	↓Proliferation ↓Apoptosis and necrosis	↑Cells in S phase arrest ↓Cells in G1 and G_2_/M phase	[108]
A431 human skin carcinoma cells	A431 cells were treated with 20–100 mg/L resveratrol for 24 hr	↑Apoptosis ↓Proliferation	↑Activation of MAPK pathway	[81]
	A431 cells were treated with 20, 50, and 100 *μ*M resveratrol for up to 72 hr	↓DNA synthesis and proliferation ↓Cells in G_2_/M phase ↓Cell cycle regulatory proteins	↓DNA-binding activity of AP-1 ↓ERK1/2 signaling pathway	[109]
Human squamous cell carcinoma cell lines	HSC2 cells were treated with both resveratrol and benzoxazinotropone at various concentrations for 48 hr	Resveratrol and benzoxazinotropone synergistically inhibited proliferation	ND	[110]
Head and neck squamous cell carcinoma cells	Head and neck squamous cell carcinoma cells were treated with 15 and 50 *μ*M resveratrol for up to 72 hr	↓Proliferation ↑Apoptosis	↑H2AX ser-139 phosphorylation	[111]
Mice	Mice with squamous cell tumor graft were gavaged orally with 10 and 50 mg/kg body weight of resveratrol for 30 days	↓Tumor weight and volume per mouse		

*Anti-inflammation*				
Keratinocytes	Cells treated with 20 ng/ml TNF-*α* for 6 hr 10 *μ*M, followed by incubation with resveratrol for additional 16 hr	↓IL-6 and MCP-1	↓Phosphorylation of I*κ*B*α*	[102]
	Cells treated with 7.5 *μ*g/mL lipopolysaccharide for 12 hr, followed by incubation with 10–50 *μ*M resveratrol for additional 12 hr	↑Proliferation ↑Apoptosis ↓IL-6, IL-8, and TNF-*α* mRNA and protein	↑miR-17 expression	[112]
	Cells treated with 50 *μ*M resveratrol 1 hr prior to addition of 2.5 *μ*g/mL lipopolysaccharide	↓IL-6, IL-8, MCP-1, and COX2 mRNA	↓EGFR-ERK signaling pathway	[91]
	Cells pretreated with 25 and 50 *μ*M resveratrol for 30 min, followed by incubation with 25 *μ*g/ml cetuximab or 2 *μ*M gefitinib for additional 3 hr	↓CCL2 and CXCL10 mRNA and protein	↓Interferon regulatory factor 1 and phosphorylated STAT1	[113]
	Cells pretreated with 50 *μ*M resveratrol for 1 hr, followed by incubation with for 10 ng/ml IFN-*γ* and TNF-*α* 24 hr	↓IL-6	ND	[114]
	Cells pretreated with 44 *μ*M resveratrol for 24 hr, followed by exposure to heat stress for 40 min	↓IL-6, IL-8, and TNF-*α*	ND	[115]
Mast cells	RBL-2H3 mast cells were pretreated with 1–25 *μ*M resveratrol for 2 hr, followed by exposure to 200 ng/ml dinitrophenyl-human serum albumin	↓IL-3, IL-4, IL-13, and TNF-*α* ↓Fc epsilon receptor I expression	↓P38-MAPK, ERK1/2, JNK	[116]
Reconstructed human skin	3D skin was treated 10 ng/ml IFN-*γ* and TNF-*α* twice a week, followed by treatment with 1% resveratrol thrice weekly for 2 weeks	↓IL-6	ND	[114]
Mice	BALB/c mouse ears were treated topically with 10 mM resveratrol 2 hr prior to DNFB challenge	↓Ear thickness ↓CD3-positive cells ↓ICAM-1, CCL2, and CXCL10 expression	↓Interferon regulatory factor 1 and phosphorylated STAT1	[113]
	Following induction of allergic contact dermatitis, NC/Nga mice were treated topically with 2.5% resveratrol or resveratrol-enriched rice extract twice weekly for 5 weeks	↓Epidermal thickness ↓Dermatitis score ↓Serum IgE ↓TEWL ↑Skin hydration	ND	[114]
	BALB/c mice were orally treated with resveratrol at a dose of 10 mg/kg body weight 1 hr prior to intravenous challenge with 200 *μ*g dinitrophenyl-human serum albumin	↓IL-4 and TNF-*α* ↓CD11b-positive cells	↓Tyk2-STAT1 activation	[116]
	Atopic dermatitis-like lesions were induced by topical applications of DNFB to the back of BALB/c mice for 5 weeks, followed by orally given resveratrol at a daily dose of 30 mg/kg body weight for 1 week	↓Dermatitis scores ↓Epidermal thickness ↓Cytokine mRNA	ND	[117]
	Atopic dermatitis-like lesions were induced by topical applications of dermatophagoides farinae to the back of NC/Nga mice for 2 weeks, followed by orally given resveratrol at a daily dose of 20 mg/kg body weight for 2 weeks	↓Dermatitis scores ↓Cytokine mRNA and protein	↓High mobility group box 1 expression	[118]
	Psoriasis-like skin lesions were induced by topical applications of imiquimod to the back of BALB/c mice, which were orally given resveratrol at a daily dose of 400 mg/kg body weight, for 7 days.	↓Erythema and scale scores ↓Skin thickness ↓Cytokine mRNA	ND	[119]

*Accelerating wound healing*				
Rats	Rats were fed with resveratrol at a daily dose of 0.5 mg/kg body weight 7 days prior to operation and continued throughout the whole experiment period	↑Collagen deposition ↑Neovascularization ↑Fibroblast maturation	ND	[120]
	Following induction of full-thickness skin wound, wound was treated topically with 225 *μ*L of 50 *μ*M once daily for 17 days	↑Epithelialization ↓Wound size ↑Collagen deposition ↑Vascularization	↑AMPK pathway and SIRT1	[121]
Mice	Immediately after wound, a wound dressing containing 0.04% resveratrol was applied to full-thickness skin wound for 10 days	↓Wound size ↑Collagen fibers ↓Inflammation	ND	[122]
	Placing scaffolds containing 5% resveratrol on the wound for 7 days	↓Wound size	↑Expression of thioredoxin-1, heme oxygenase-1, and VEGF	[123]
Diabetic models	0.5% resveratrol ointment was applied to wound area in diabetic rats once daily for 21 days	↓Wound size	↑Activity of antioxidant enzymes	[124]
	10 *μ*M resveratrol was applied to the cutaneous wound area in diabetic mice, and wound healing was assessed 7 days later	↓Wound size ↑Endothelial cell proliferation	Sirt1 activation	[125]
	Fourteen days after topical application of resveratrol (0.1 mg/ml) to wound area in diabetic rats once, wound healing was assessed	No benefit		[126]

Abbreviations: ND, not determined; AQP3, aquaporin 3; SOD, superoxide dismutase; MDA, malondialdehyde; GSH-Px, glutathione peroxidase; GST, glutathione S-transferase; GSH, reduced glutathione; NQO, NAD(P)H:quinone oxidoreductase; ROS: reactive oxygen species; CYP1A1, cytochrome P540 family 1 subfamily A member 1; IKK*α*, Ι*κ*B kinase *α*; CYP1B1, cytochrome P540 family 1 subfamily B member 1; COX, cyclooxygenase; ODC, ornithine decarboxylase; NRF2, nuclear factor erythroid 2-related factor 2; ERK: extracellular signal-regulated kinase; MAPK, mitogen-activated protein kinase; JNK, c-Jun NH2-terminal kinase; TPA, 12-O-tetradecanoyl13-phorbol acetate; DNFB, 2,4-dinitro-1-fluorobenzene; NOS, nitric oxide synthase; VEGF, vascular endothelial growth factor; AMPK, adenosine monophosphate‐activated protein kinase.

## Supplementary Materials

Supplementary Materials Supplemental Table 1: content of resveratrol in some plants and foods/beverages.
