# Supplementary material for: Role of Resveratrol in Regulating Cutaneous Functions
Source: Evid Based Complement Alternat Med. 2020 Apr 14;2020:2416837. doi: 10.1155/2020/2416837 (PMC7180429; doi:10.1155/2020/2416837)
Supplement: Supplementary Materials — Supplemental Table 1: content of resveratrol in some plants and foods/beverages. [file 2416837.f1.pdf]

Supplemental Table 1. Content of Resveratrol in Some Plants and Foods/Beverages

Plants/foods	Content			References
	trans-resveratrol	*cis*-resveratrol	Total	
**Grapes**				
Merlot[a]	0.5 ± 0.0	ND	0.5 ± 0.0	1
Cabernet Sauvignon[a]	0.5 ± 0.0	ND	0.5 ± 0.0	1
Primitivo[b]	13.9 ± 0.4	ND	13.9 ± 0.4	2
Negroamaro[b]	3.6 ± 0.1	ND	3.6 ± 0.1	2
Susumaniello[b]	63.0 ± 0.9	ND	63.0 ± 0.9	2
Uva diTroia[b]	4.6 ± 0.06	ND	4.6 ± 0.06	2
Malvasia Nera[b]	48.5 ± 1.0	ND	48.5 ± 1.0	2
Aglianico[b]	61.1 ± 0.8	ND	61.1 ± 0.8	2
Cesanese[b]	8.16 ± 0.02	ND	8.16 ± 0.02	2
Merlot Moscato[b]	9.2 ± 0.2	ND	9.2 ± 0.2	2
Alphonse Lavalle´e[b]	3.89 ± 0.01	ND	3.89 ± 0.01	2
Rootstock[a] (Beta)	1.59	ND	1.59	3
Rootstock[a] (Shanhe)	7.05	ND	7.05	3

Rootstock[a] (Zhi)	6.21	ND	6.21	3
Rootstock[a] (Berlandier Resseguier 2)	4.04	ND	4.04	3
Rootstock[b] (Dog Ridge)	3.87	ND	3.87	4
California Table Grapes[a] (Flame Seedless (skin))			1 ± 0.3	5
California Table Grapes[a] (Black Corinth (skin))			<0.1	5
**Tomato Skin[b]**				
MicroTom tomato	15.3	2.71	19.4	6
Beefsteak	ND	ND		6
Uglyripe	0.38	ND	0.38	6
Heirloom	1.75	0.11	1.86	6
Plum Tom	0.34	ND	0.34	6
**Juices[c]**				
Red grapes juice 100%	40.0 ± 0.60	ND	40.0 ± 0.60	2
Grape juice made from concentrate 99%	0.27 ± 0.01	ND	0.27 ± 0.01	7
Organic grape juice made from concentrate	ND	ND	ND	7
Red grape Merlot juice 100%	0.16 ± 0.01	ND	0.16 ± 0.01	7
Red grape juice 65%, pomegranate juice 25%, blackcurrant juice 10%	ND	ND	ND	7

Grape juice 50%, pomegranate juice 50%	0.36 ± 0.02	ND	0.36 ± 0.02	7
**Peanuts**				
Runner[b] (White's runner)			0.07 ± 0.03	8
Spanish[b] (Spanette)			0.13 ± 0.11	8
Spanish[b] (Pear)			0.11 ± 0.09	8
Spanish[b] (GA 207-3-4)			0.06 ± 0.07	8
Spanish[b] (PI-337396-FAV70)			0.02 ± 0.02	8
Valencia[b] (Kalasin 1*)			1.4 0.04	8
Valencia[b] (Kalasin 2*)			1.57 0.82	8
Valencia[b] (Konkaen*)			1.2 0.57	8
Valencia[b] (Konkaen 4*)			2.88 1.23	8
Virginia[b] (NC-18016)			0.31 ± 0.15	8
Virginia[b] (Early Bunch)			0.13 ± 0.19	8
Virginia[b] (NC-17291)			0.05 ± 0.07	8
Boiled peanuts[a]	5.1 ± 2.8	ND	5.1 ± 2.8	1
Peanut butter[a]	0.3 ± 0.1	ND	0.3 ± 0.1	1
Whole Peanut sprout[b] (Germination day 0)	Not Determined		3.7 ± 0.6	9
Whole Peanut sprout[b*] (Germination day 1)	Not Determined		6.0 ± 0.9	9

Whole Peanut sprout[b] (Germination day 2)	Not Determined		17.7 ± 3.2	9
Whole Peanut sprout[b] (Germination day 3)	Not Determined		19.9 ± 2.5	9
**Wines**				
Pinot Noir, 1994 (California)[c]	10.57 ± 0.6	7.46 ± 0.09	18.03	1
Cabernet Sauvignon, 1996 (Bulgaria)[c]	6.72 ± 0.10	5.20 ± 0.16	11.92	1
Merlot, 1994 (Chile)[c]	0.48 ± 0.01	1.52 ± 0.05	2.0	1
Merlot, 2000(Brazilian) [c]	3.04	7.93	10.97	10
Merlot, 1999(Brazilian) [c]	ND	12.25	12.25	10
Merlot, 2002 Giacomin commercial(Brazilian) [c]	5.34	21.4	26.74	10
Merlot, 2002 Giacomin in tank(Brazilian) [c]	4.86	22.99	27.85	10
Merlot, 2002(Brazilian) [c]	2	4.9	6.9	10
Merlot 2003 microwinemaking(Brazilian) [c]	1.77	23.23	25	10
Merlot (Italian) [c]	2.24	2.09	4.33	11
Cabernet sauvignon 2003 microwinemaking(Brazilian) [c]	ND	4.16	4.16	10
Cabernet sauvignon 2002 Giacomin(Brazilian) [c]	2.41	1.70	4.11	10
Cabernet sauvignon 2000(Brazilian) [c]	ND	3.04	3.04	10
Cabernet-sauvignon 2001 (Brazilian) [c]	2.06	7.02	9.08	10
Cabernet Sauvignon, 1995 (California) [c]	0.53 ± 0.01	0.45 ± 0.01	0.98	1

Cabernet franc 2001(Brazilian) [c]	2.27	8.43	10.70	10
Tannat 2000(Brazilian) [c]	ND	1.91	1.91	10
Monovarietal white wines (Portugal) [c]	0.6	0.4	1.0	12
Fortified wines (Portugal) [c]	0.8	0.1	0.9	12
Monovarietal red wines (Portugal) [c]	1.0	2.6	3.6	12
Blended red wines (Portugal) [c]	1.5	2.1	3.6	12
Blended white wines (Portugal) [c]	0.5	0.2	0.7	12
Red wines (France) [c]	3.0	2.6	5.6	12
Vintage 1990[c]	1		1	13
Vintage 1995	15 ± 3[d]		15 ± 3	13
Vintage 1997	48 ± 5[d]		48 ± 5	13
**Itadori Root**				
Commercial root[a]	523 ± 1	ND	523 ± 1	1
Itadori tea[c]	0.68 ± 0.01	ND	0.68 ± 0.01	1
Hairy root cultures of Ligularia fischeri[b]				14
**Berries**				
Raw highbush Michigan blueberry	140.0 ± 29.9[e]		140.0 ± 29.9	15
Raw highbush British Columbia blueberry	ND		ND	15

Raw rabbiteye Mississippi blueberry	ND	ND	15
Raw lowbush "wild" Nova Scotia blueberry	56.2 ± 15.7[e]	56.2 ± 15.7	15
Raw Polish bilberry	71.0 ± 15.0[e]	71.0 ± 15.0	15

[a]Data expressed µg/g fresh weight, mean ± SE; [b]Data expressed µg/g dry weight, mean ± SD; [c]Data expressed as mg/litter, mean ± SE; [d]Data expressed as mg/litter, mean ± SD; [e]pmol/g, mean ± SD; *1-day germination; ND: not detected.
